# An Emotional Agent for Moral Impairment Rehabilitation in TBI Patients

**DOI:** 10.3389/fpsyg.2020.01102

**Published:** 2020-06-30

**Authors:** Eleonora Ceccaldi, Rossana Damiano, Cristina Battaglino, Valentina Galetto, Marina Zettin

**Affiliations:** ^1^InfoMus Lab, DIBRIS, University of Genoa, Genoa, Italy; ^2^Dipartimento di Informatica, Università di Torino, Turin, Italy; ^3^Centro Puzzle, Turin, Italy

**Keywords:** narrative, emotion understanding, moral emotions, social cognition, traumatic brain injury, computational models, models of emotion

## Abstract

The ability to identify the emotions of others is a key component of what is known as social cognition. Narratives exploit this mechanism to create an emotional bond with the characters and to maintain the engagement of the audience throughout the story. In this paper, we illustrate a case study in emotion understanding in stories that exploits a computational agent to explore emotion impairment in a group of traumatic brain injured people. The study focuses on moral emotions, aiming to investigate the differences in moral functioning that characterize traumatic brain injured patients. After comparing the understanding of the moral and emotional facets of the agent's behavior in traumatic brain injured patients and in neurologically intact controls, slight–yet meaningful–differences were observed between the two groups. We describe the test methodology and results, highlighting their implications for the design of rehabilitation applications based on virtual agents.

## 1. Introduction

Emotions play a crucial role in stories, as stated by scholars for centuries, from philosophy (Aristotle, 2013) and psychology (Bruner, 1991) to narratology (Giovannelli, 2009). In Plato's Ion, the rhapsode Ion describes his ability to evoke emotional states in the audience: “For I look down upon them [the spectators] from the stage and behold the various emotions of pity, wonder, sternness stamped upon their countenances when I am speaking.” (Plato and Jowett, 1924 as cited in Damiano et al., 2019). In narratives, emotions, and moral emotions in particular, take a leading role in engaging the audience, ensuring their involvement throughout the plot, from rise to climax to resolution (Olson, 1961; Giovannelli, 2009).

The ability to read and understand the emotions of characters is crucial to grasping the meaning of a story. Such ability relies on what is known as *social cognition*. Social cognition (Cassel et al., 2019) is an umbrella term indicating a set of processes from the ability to read social cues both from the self and from others and understanding emotions, beliefs, and behavior to the capability of generating appropriate responses to social cues. Traumatic brain injuries (here TBI), like other neurological conditions, lead to impairments in social cognition (Bibby and McDonald, 2005) as well as in controlling behavior and properly displaying emotions (Roberts et al., 2019).

Several studies point toward a relationship between social cognition and social behavior following TBI (Milders, 2019). Although addressing social impairments is a fundamental step toward full recovery (Kelly et al., 2017), testing tools for social cognition after TBI are often underutilized in clinical practice (Kelly et al., 2017).

Here, we investigate the perception of emotions in narrative scenarios by TBI individuals involved in effective cognitive rehabilitation but yet impaired, to some extent, in their moral and emotional functioning (i.e., inability to empathize, difficulties in understanding their own emotions, etc.). More specifically, we focus on the understanding of narrative situations involving moral emotions. In fact, authors have claimed that moral impairments following TBI should gain more attention in research (McDonald, 2013), as moral capabilities greatly affect the quality of life of patients and of their caregivers (Saint-Jean et al., 2019). The use of narrative scenarios is two-fold: on the one side, thanks to the universally acknowledged capability of narrative to convey values in a compact and effective form for human cognition (Bruner, 1991), they represent a suitable format for presenting moral dilemmas to the patients; on the other side, these scenarios can afford the creation of applications for training and rehabilitation that prompt the patients to reason about the emotions of characters in fictional stories. Our work leverages a computational agent model to explore the difference in the understanding of moral actions and emotions in narrative scenarios among TBI patients and neurologically intact individuals.

Aimed at generating human-like, believable behaviors, computational agents rely on cognitively inspired architectures where emotions and deliberation affect each other to replicate the complex interaction of rational and emotional components in humans (Marsella et al., 2010; Lisetti and Hudlicka, 2014). Since the output of the agent can be compared with the predictions of the audience about what the character will feel and do, virtual agents provide a stable, verifiable framework for the design and the implementation of experiments in story understanding.

In addition, the agent can afford the design of characters who behave and feel according to specific psychological theories or can be set to standard functioning to support the creation of rehabilitation applications (Habonneau et al., 2012; Chauveau et al., 2018).

In order to study the moral emotional impairment of TBI patients through stories, this study employs narrative scenarios whose characters are replaced by a computational agent that encompasses moral values and emotions, the *Moral Emotional Agent* or *MEA* (Battaglino et al., 2013).

Our methodology, previously sketched in Ceccaldi et al. (2016), is aimed at comparing the differences in the understanding of emotions, focusing on moral emotions between traumatic brain injured patients and neurologically intact controls. Drawing from the experiments described by Battaglino and Damiano (2014a), where the emotions generated by the agent were compared with the emotions ascribed to the narrative character by the human users, we compare the emotions generated by the agent with those identified by the test and control groups to investigate the differences between the two groups. The results confirm the role of emotions in the understanding of narratives and highlight the impairment of TBI patients in the understanding of moral emotions. The advantage of our approach is two-fold: on the one side, new testing scenarios can be easily generated by submitting new plots to the computational agent; on the other side, the agent can be straightforwardly employed to implement virtual characters to train the patients through stories. Differently from abstract dilemmas, stories possess the unique quality of engaging the audience, and this provides a natural candidate for creating effective training tools. This is also in line with the use of virtual characters in applications for health care and medicine, where the affective dimension has been recognized as a main requisite for establishing successful and effective relationships with the patients (Calvo et al., 2014).

### 1.1. Related Work

Behavioral consequences of traumatic brain injury can be a greater burden for caregivers of brain damaged individuals than physical consequences of the injury (Bornhofen and Mcdonald, 2008). Despite being relatively well documented, the impairments underlying the negative social outcomes occurring after TBI are far from being fully understood (Milders et al., 2008). Moreover, there is currently a lack of diagnostic tools to assess the changes in social behavior and cognition (Cattran et al., 2016). The tools proposed in the literature have addressed several aspects of this area while also leveraging different testing tools and methodologies, such as emotion recognition (in terms of facial expressions), theory of mind, sensitivity to social cues, complex language (inference, humor), empathy, understanding of paralinguistic cues, social interaction and social anxiety (Cattran et al., 2016). A thorough review of testing tools for social cognition after TBI goes beyond the scope of this study. Most used testing methodologies have been recently described by Milders (2019). Nonetheless, works on emotion understanding in stories might help the reader better fathom our study on narrative scenarios. The Emotional Inferencing From Story (EIST) proposed by Neumann et al. (2015) assesses the ability to understand emotions of others from contextual cues. The purpose is to measure emotion understanding when non-verbal cues are unavailable; to do this, patients were presented with short stories and asked to evaluate how the character in that story was feeling. When compared with neurologically intact individuals, TBI participants scored significantly lower, showing EIST validity in assessing impairments following TBI. Similarly, Saint-Jean et al. (2019) illustrate how social cognition deficits in TBI patients can be effectively assessed through narrative scenarios. For instance, they describe the social problem solving task, a test made up of 10 written stories depicting a character facing a social problem. In the test, participants had to detect and understand the problem and to identify its key components. Furthermore, the task required them to propose solutions to the problem or to evaluate solutions that were already presented in the scenario. Patients scored significantly lower than healthy controls in the social problem task. What is more, when it comes to training such ability to understand the emotional content of stories, research has shown that it did positively impact social behavior according to caregivers' ratings (Radice-Neumann et al., 2009). Radice-Neumann et al. trained TBI patients to infer emotions from contextual cues portrayed in stories, and to make connections between these stories and personal events. The training relied on short stories; while reading the scenarios, participants also had to take contextual cues of emotional features (i.e., characters' wants, expectations, and behavior) into account and relate the story to personal lives. After reading each scenario, participants were asked to select the strongest emotion they believed the character was feeling. Feedback was given for incorrect responses. When the correct option was provided, participants were asked why it was correct, how they would have felt in that situation, if they ever found themselves in similar circumstance and to state a life event that had made them experience that emotion. The training resulted in participants being more comfortable in reporting their emotions and better able to handle their emotions in challenging situations. Caregivers of participants reported improvements in attitude and ability to communicate feelings.

## 2. Modeling Story Characters With the MEA Agent

In the last decade, the use of virtual agents in health care has been explored in different domains, spanning from nursing (Bickmore et al., 2012) and counseling (LeRouge et al., 2015) to training of people with autism (Burke et al., 2018) and assistance to elderly and cognitively impaired people (Yaghoubzadeh et al., 2013; Chauveau et al., 2018). Virtual agents can be implemented on different devices in an non intrusive way, thus guaranteeing portability and continuity in therapies, and can be personalized to meet the needs of specific patients or groups of patients. In particular, the integration of an affective component in virtual agents has attracted the attention of scholars, since it opens to the creation of empathetic virtual agents, more natural and believable in the interaction with the user (Lisetti and Hudlicka, 2014).

### 2.1. The MEA Model

The core reference model for creating virtual agents is provided by the widely acknowledged Belief Desire Intention (BDI) model (Bratman, 1987; Cohen and Levesque, 1990). The BDI model, informed on Dennet's notion of “intentional stance” (Dennett, 1987), is suitable to simulate the intentions behind the behavior of human agents, and as such can be effectively employed to create virtual agents that interact with human users. Following this model, the MEA agent (Battaglino et al., 2013) features a set of goals, or *desires*, composing the motivational component of the agent; the *beliefs* of the agent are formed by a representation of the world, continuously updated through perception, and by the knowledge about how actions can be planned and executed in the world to achieve the agent's goals; the agent's commitment to execute action plans bridges the gap between the agent's abstract goals and its practical *intentions*. The MEA model integrates in the BDI model an emotional component based on the cognitive theory of emotions proposed by Ortony et al. (1988) (here OCC). In MEA, a goal is associated with an *importance of success* and an *importance of failure*, and with three different set of conditions: *adoption conditions, success conditions*, and *failure conditions*. A goal becomes an *active intention* when the agent believes that one of the adoption conditions of a goal is true in the world; at this point, the agent starts the deliberation phase, trying to find plans to achieve the goal's *success conditions* and dropping those that have been achieved or whose *failure conditions* have become true. Psychology shows that decision-making relies on preferences that vary with the subjective utilities of anticipated outcomes, weighted by their probabilities (Angie et al., 2011) and that decision-making and moral judgment are related to how people combine desires, personal values, and expectation to choose a course of action. So, the agent, after devising a set of plans to achieve its goals, ranks them by combining the achievement of goals (measured through their *importance of success* and the *importance of failure*) with a measure of its own emotional well-being (*Expected Emotional Reward, EER*) and becomes committed to the plan with the best trade-off between positive and negative emotions, then starts to execute it. After monitoring the effects of the execution on the state of the world (the plan might have succeeded or not, other events may have occurred), the agent eventually updates its beliefs and emotional state accordingly.

A characterizing feature of the MEA model is given by the explicit acknowledgement of *values* in the appraisal of emotions. Values (or “standards” in OCC terms) are the moral drive of the agent (Fraassen, 1973; Dehghani et al., 2008), which binds its behavior to a moral dimension and enables it to morally appraise the behavior of self and others, thus eliciting moral emotions. In the MEA agent, each value holds a *priority* that indicates the importance of the value for the agent, and a set *violation conditions*. When one of the violation conditions of a value is true in the state of the world, the value is *at stake* and will originate a goal to bring it back to balance. In the reasoning cycle of MEA, the appraisal of emotions occurs twice: the first time, during the deliberation phase (*Anticipatory Emotional Appraisal*), to assess the consequences of the agent's options on the agent's emotional state; the second time, after assessing the changes occurred in the world (*Emotional Appraisal phase*), to generate the agent's actual emotional state, according to the following schema:

1. **Value Monitoring**: If the agent believes the condition of a value to be true in the current situation, then the value is *at stake*.
2. **Goal Formation**: Goals whose adoption conditions hold in the belief base are adopted and become *active intentions*; they include value-based goals, motivated by the values at stake.
3. **Emotional Anticipatory Appraisal**: After computing the *expected emotional reward* (EER) of every goal from its associated plans, the agent chooses the optimal plan; in this phase, the agent “feels” only anticipatory emotions.
4. **Execution**: The agent starts the execution of the next action of the chosen plan.
5. **Monitoring**: The agent appraises the world and updates her beliefs about it, including the status of its goals and values.
6. **Emotional Appraisal**: depending on the updates observed in the state of the world (goals achieved or failed and values re-established or at stake) the agent feels certain emotions.

Consider, for instance, an agent who desires a chocolate treat, but hasn't got one. Having learnt that another agent has a chocolate candy (adoption condition), the goal to eat the chocolate candy becomes the agent's active intention (*Goal formation*). So, the agent starts planning how to achieve her goal, eventually devising two plans: asking the other agent to give her the candy, or stealing the candy. The agent appraises the effect of each plan on her goals and values, and ranks the plans according to their expected emotional reward (*Emotional Anticipatory Appraisal*). Depending on the goal and value structure of the agent (whether she is inclined to follow the rules, optimistic about others, etc.), the ranking of plans will vary. Eventually, the agent selects a plan and executes it (*Execution*), monitors its effects (*Monitoring*) and feels emotions based on its outcome: gratitude, anger, disappointment, satisfaction, etc. (*Emotional Appraisal*). When the reasoning cycle starts again, the agent might realize one of her values to be at stake (*Value Monitoring*): for example, because she has put at stake her honesty due to her desire for chocolate.

### 2.2. Emotional Appraisal in MEA

The reference theory of emotions in the MEA agent is the OCC theory (Ortony et al., 1988), chosen for its capability to match the emotional range of characters in the perception of the audience (Lombardo et al., 2015). In the OCC model, events are appraised based on the their desirability for the agent's goal, self, and others' actions are appraised based on their compliance with the agent's moral standards, objects are appraised based on the agent's specific attitudes toward them.

**Figure 1:**
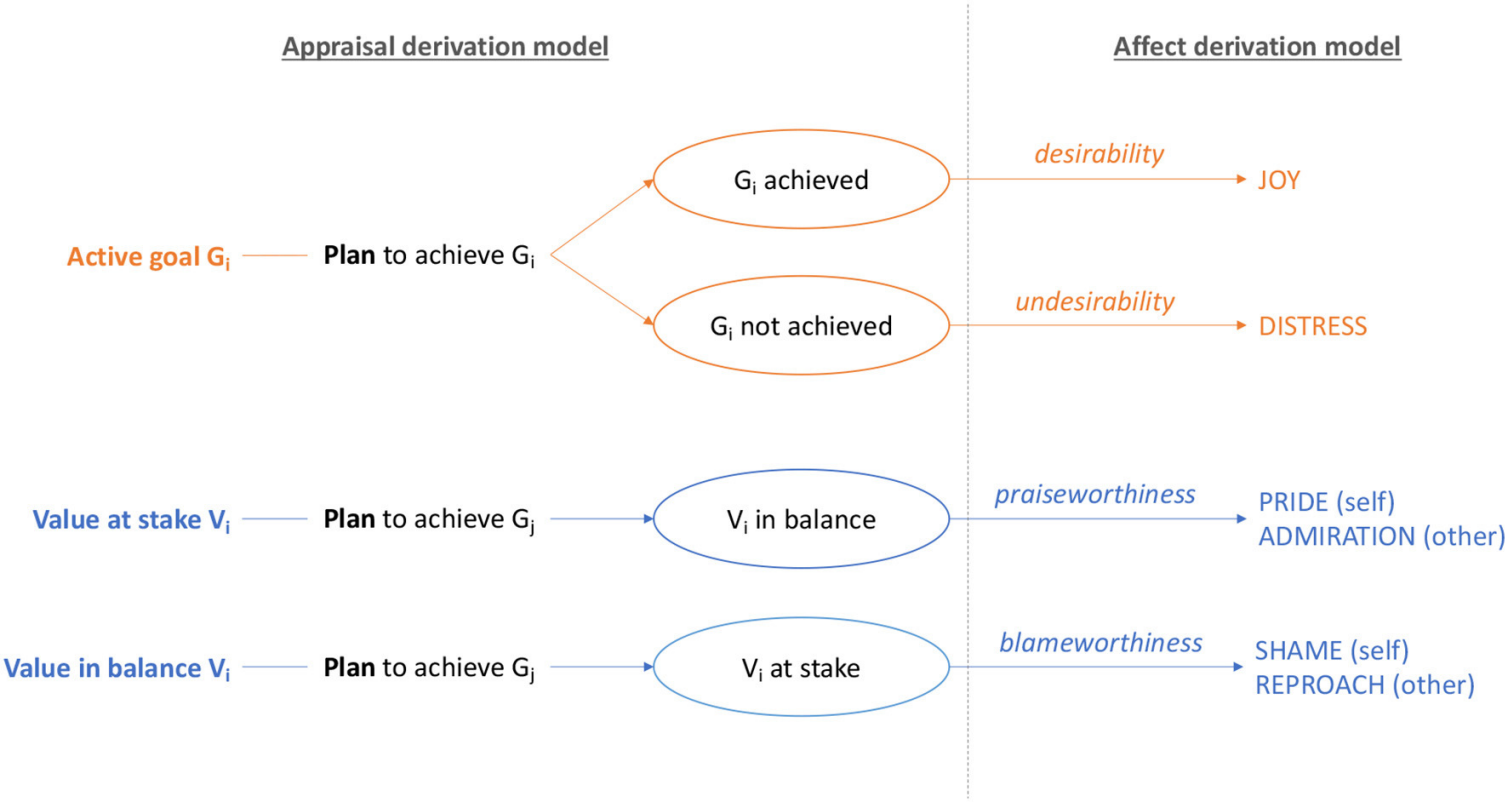
Emotional appraisal and affect generation in the MEA model. Other appraisal variables (importance of goals, priority of values and plan length) do not determine the affect type, but only the intensity, and are not represented in the figure.

Following the general framework established by Gratch and Marsella (2004), the generation of the emotional states in MEA is a two-step process:

First, the appraisal generates a set of *appraisal variables*, such as the desirability and probability of an event, each associated with some intensity; in the *affect derivation* process, emotional states are activated based on the appraisal variables. In practice, when an agent's goal is achieved (or not achieved), the appraisal process generates a *desirability* variable (or an *undesirability* variable); when an agent's value is put at stake (or brought back to balance) by the execution of some action, the appraisal process generates a *blameworthiness* (or *praiseworthiness*) variable; the probability that an event occurs or that a agent's plan succeeds generates a *likelihood* variable. Based on the appraisal variables, the affect generation process generates emotions according to the following rules (see Figure 1):

- **Joy** (or **Distress**) if a *desirable* (or *undesirable*) appraisal variable is generated;
- **Pride** (or **Shame**) if *praiseworthy* (or *blameworthy*) appraisal variable is generated and the responsibility is *self-caused*;
- **Admiration** (or **Reproach**) if *praiseworthy* (*blameworthy*) appraisal variable is generated and the responsibility is *other-caused*.

When the same situation is appraised as both an action and a non-intentional event, appraisal variables for both values and goals are generated, thus eliciting compound emotions: Gratification (Joy and Pride), Gratitude (Joy and Admiration), Remorse (Distress and Self-Reproach), Anger (Distress and Reproach). If a *likelihood* appraisal variable has a high (or low) value, Hope (or Fear) are generated. The *intensity* of emotions depends on the multiplicative relationship between the importance of values and goals, the effort (i.e., the length of the plan) and the probability of success of the plan.

As a consequence of the anticipatory emotional appraisal, the behavior of the agent is compelled by its moral values through anticipatory emotions, a feature that makes it especially suitable to model moral dilemmas in stories (Williams, 2006), where introspection plays a prominent role. Also, the agent model guarantees that the dynamics of the agent's deliberative and emotional processes can be traced and exposed to the users, in line with the requisite of transparency and explainability required by the principles of trustworthy AI ^1^.

For example, consider again the agent who desires chocolate, but can only steal it from another agent or ask for it. Assuming that stealing puts the agent's honesty at stake, the agent's behavior depends on the anticipatory emotional appraisal: if honesty is of minimal importance for the agent (i.e., it has a low priority), negative emotions such as the shame generated by the act of stealing, or the fear to fail a risky plan, will be largely compensated by the satisfaction of eating the candy, and agent will end up stealing; on the contrary, if honesty is very important for the agent (i.e., it has a high priority), the expectation of shame will retain her from stealing, unless she is really hungry. Notice that even if the agent eventually decides to steal, this won't stop her to feel shame, possibly with a low intensity, and mixed with satisfaction or disappointment depending on whether she actually obtained the chocolate candy.

### 2.3. Validating the MEA Model

In order to validate the MEA model, Battaglino and Damiano compared the behavior and the emotions generated by the MEA agent with the predictions of human participants in a set of narrative scenarios (Battaglino and Damiano, 2014a,b). Following the suggestions of a drama expert, the scenarios for both experiments were taken from well-known literary works, thus lifting the experiment design from the task of inventing new and potentially controversial narrative situations. The original experiments included two tests, structured as simple games. The Actor Studio (Battaglino and Damiano, 2014a) test was designed to validate the role of emotions in the agent's deliberation: given the character's scale of moral values, participants were asked what course of action the character would choose, and what emotions she/he would feel, as if they were practising at the well-known “Actor's Studio” following the Stanislavski's acting method. In order to bypass the participants' previous knowledge of the literary works, the scenarios were re-written with different characters and actions but keeping the interplay of characters' goals and values unmodified. The test included 3 narrative scenarios (see Appendix 1.1) where the main character's options (plans in agent's terms) were generated by the MEA agent given the character's goals and values (see section 2.1). After receiving a description of the character's goals and values, each participant was asked to choose the most suitable option for the character.

The Audience Studio (Battaglino and Damiano, 2014b) test was aimed at validating the interplay of values and emotions in the agent: all participants were exposed to the same characters' behavior with two different conditions—with and without moral emotions—and had to assess their adequacy, as if they were assessing the performance of actors interpreting the characters. The test included 4 narrative scenarios (see Appendix 1.2) where the main characters were implemented by the MEA agent (Battaglino et al., 2013; Cristina, 2015). Differently from the Actor Studio, where alternative options were generated to create the user choices, in this test only the actual behavior of the character in the literary work was generated, with and without value-based emotions. Since the participants' knowledge of the characters' actions was not relevant here, the original characters and actions were maintained.

The results, discussed in Battaglino and Damiano (2014a,b), showed that the expectations of the testers matched the predictions of the model: for the Actor Studio, the participants attributed to the character the course of behavior and the emotions predicted by the model (Battaglino and Damiano, 2014a); for the Audience Studio, the emotional states including moral emotions were evaluated as more complete and believable (Battaglino and Damiano, 2014a,b).

## 3. Experimental Methodology

In this study, both tests described in Battaglino and Damiano (2014a) were used to explore emotion understanding in narrative in TBI patients. In previous studies, the expectations of neurologically intact individuals on characters' behavior and emotions have been demonstrated to match predictions made by modeling characters as MEA agents (Battaglino and Damiano, 2014a,b). Therefore, this study could leverage such testing tools to compare MEA agents' behavior and emotions with TBI participants' expectations and predictions.

### 3.1. Participants

The clinical sample was recruited from “Puzzle" rehabilitation center in Turin (Italy)^2^.

The clinical sample was made up of 14 TBI individuals (mean age 40.3), involved in neuropsychological rehabilitation at the center from 1 to 13 years (mean 5.5). Being in its exploratory phase, our study involved an heterogeneous sample, with participants having different cognitive profiles and clinical histories, with lesions being localized mostly (but not exclusively) in the frontal area. However, they were all recruited according to their ability to execute the testing procedures, to read and to verbally understand the scenarios thoroughly, along with having difficulties in social cognition (e.g., emotion understanding, empathy). In fact, only the patients that were deemed able to complete the tasks by the center neuropsychologists were invited to participate. Moreover, prior the experiment, each participant had the possibility to familiarize with the experimenter and with the testing procedure, to avoid stress and discomfort. As completing the task required full recovery of specific cognitive abilities (i.e., linguistic and narrative abilities to read and understand the plot), recruiting participant was challenging, resulting in a small sample. Before starting the tests, two TBI patients with a cognitive profile similar to that of participants took part in the study in order to test if their cognitive impairments (i.e., reading difficulties, attention deficits) would result in the task being too difficult or impossible to complete. Secondly, the methodologies were slightly adapted to the needs of the participants; for example they were made shorter (as patients were very slow in reading, often taking longer than 60 minutes for each test). After this, the Actor Studio and the Audience Studio testing methodologies were presented to a new sample made of TBI patients and healthy controls, in order to compare the groups. The control group was a convenience sample including neurologically intact individuals (i.e., people that declared to the experimenters never suffering from TBI), matching each patient for age and gender.

### 3.2. Actor Studio Testing Methodology

**Figure 2:**
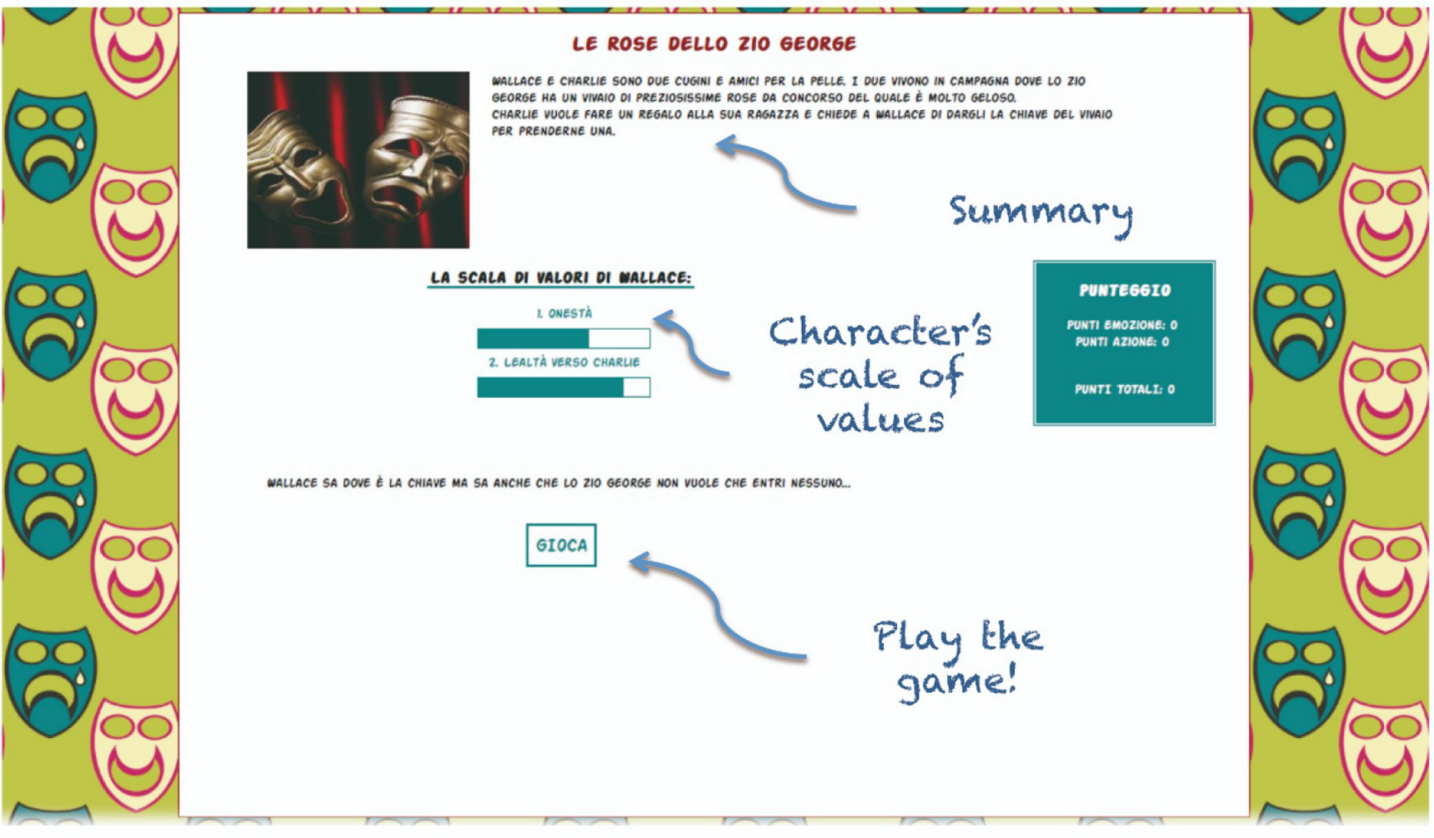
The screenshot of the online interface of the Actor Studio test (in Italian).

The experiments were conducted online. For each scenario (see Appendix 1.1), a short text introduced the character and her/his values, then the narrative situation was illustrated. Figure 2 shows a screenshot of the page in which the narrative scenario is illustrated. The character's scale of values was presented to the participant not in a numerical format, but with a figurative scale, in order to make the priorities of the values as clear and understandable as possible. The task of identifying the expected course of action and emotions for the character was presented to the participants as a game: participants were asked to pretend they were practicing identification in an acting class, trying to adopt the point of view of the characters as actors following the Stanislavski's acting method. By pressing the “play” button (bottom of the page, Figure 2), the two alternative actions generated by the MEA agent were presented to the participant, who then had to indicate a set of possible emotions (taken from the set of the 12 emotion types included in the MEA model, see section 2.2).

### 3.3. Audience Studio Testing Methodology

The experiment was conducted online. It was made up of the 4 narrative scenarios illustrated in Appendix 1.2, taken from well-known literary works (namely Hamlet, The Count of Montecristo, Thérèse Raquin, and The Vicomte of Bragelonne). After a brief description of the narrative situation, participants were presented with a dialogue between the characters involved in the scenario. The dialogues were extracted from movies or books for more immediacy. After this, characters' emotions were described through text labels (e.g., “Hamlet feels reproach toward Ophelia”). Participants were then asked to select, from a list, those emotions they would feel in a similar situation (again, the set of 12 emotions from OCC model). Differently from the Actor Studio, subjects had to evaluate both the emotions of the protagonist and of the other character. Whereas, the Actor Studio testing methodology allowed scores for each scenario to be related to the same participant, the results for the Audience Studio methodology could only be measured separately for each scenario. Although simplified to meet the needs of TBI patients, the Audience Studio testing procedure took more time than the Actor Studio to be completed. Scenarios are in fact longer to be read and understood and the testing procedure also contains items we eventually decided not to take into account (e.g., the participants' evaluation of the characters' attitude and behavior). For this reason, TBI participants were sometimes unable to complete the testing procedure all at once, as their daily schedule in the rehabilitation center only allowed them to participate in the testing for about 45–50 min. Therefore, some participants completed the test through several sessions, resulting in scores being impossible to be related to the same participant. Regrettably, the system did not record the experiment time, thus causing sessions taking place on the same day to be indistinguishable.

By contrast, the Actor Studio methodology required the participants to choose a nickname for their performance. This, along with the testing procedure being simpler and therefore shorter, allowed every single session data to be related to its tester and with the right scenario. The aforementioned characteristics of the testing methodologies allowed us to compare scores between groups for both methodologies, but a deeper analysis of the scores and of their correlation with the clinical data was possible only for the Actor Studio test.

## 4. Measures and Results

This section illustrates the measures employed to compare the performance of the two groups along with the results, discussed in section 5.

### 4.1. Measures

Data were analyzed through SPSS Statistics 24 (IBM). Sample size was *n* = 13 for TBI patients and *n* = 11 for the control group (as a consequence of participants drop-out). One neurologically intact participant was not included in the analysis, as his scores were significantly lower than other participants' (i.e., Action score and Emotion total score both for moral and non moral emotions being equal to 0). Participants' responses were turned into scores in order to perform comparisons between groups. As mentioned before, for the Audience Studio methodology only, scores were measured separately for each scenario.

For both methodologies, scores were calculated as follows:

- Emotion total score (ETS): score = 1 each time participants would choose the same emotion predicted by the MEA model;
- Moral emotion score (MES): score = 1 each time participants would choose the same emotion predicted by the MEA model; only for moral emotions, being them admiration, contempt, gratification, gratitude, pride, shame, anger, blame;
- Non moral emotion score (N-MES): score = 1 each time participants would choose the same emotion predicted by the MEA model; only for non moral emotions, being them joy, sadness, hope, and fear;
- Error total score (ERTS): score = 1 each time participants both would choose an emotion in disagreement with MEA model and would miss indicating those predicted by the model;
- Moral emotion error score (MERS): score = 1 each time participants both would choose an emotion in disagreement with MEA model and would miss indicating those predicted by the model; only for moral emotions;
- Non moral emotion score (N-MERS): score=1 each time participants both would choose an emotion in disagreement with MEA model or would miss indicating those predicted by the model, only for non moral emotions.

In the Actor Studio, participants were asked to predict the action characters would choose according to their moral values. Therefore, for this methodology only, we took a further measure into account:

- Action total score (ATS): score=1 each time participants could guess the agents' action according to MEA model.

For each score, means were compared using Mann–Whitney test, suitable for different-sized samples. As our aim was to compare TBI participants' similarity with the MEA agents with that of neurologically intact individuals, we also used the Simple Matching Coefficient statistic to evaluate the performance of the participants (Sokal and Sneath, 1961). In our view, this statistic allows a better comparison between the model and the participants' choices, as it takes into account also the emotions *not* predicted by the model, and those *not* selected by the participants. Considering the MEA Model emotion set as an array of emotions (some included and some not) and the participants choices as an array of selected and unselected emotions, the Simple Matching Coefficient (SMC) was calculated as follows:

(1) $$\begin{array}{r} SMC=\frac{M_{00}+M_{11}}{M_{00}+M_{01}+M_{10}+M_{11}} \end{array}$$

Where:

- *M*_00_ are the emotions not predicted by the model and not selected by the participant;
- *M*_11_ are the emotions predicted by the model and selected by the participant;
- *M*_01_ are the emotions not predicted by the model selected by the participant;
- *M*_10_ are the emotions predicted by the model not selected by the participant.

After this, SMCs for each scenario were compared between groups for both methodologies, through Mann–Whitney. We measured global SMCs, calculated on complete emotion sets and moral SMCs, considering moral emotions only. Furthermore, we performed Spearman correlation analysis to assess the relationship between time spent in the rehabilitation center (rehabilitation duration, in years) and the global Simple Matching Coefficient for the Actor Studio methodology, calculated on all three scenarios. Moreover, we investigated the relation between the time distance from the traumatic event (in years) and the aforementioned variable.

### 4.2. Results

#### 4.2.1. Results Actor

In the Actor Studio, participants were asked to select which among two alternative courses of action the characters in each scenario would choose, according to their moral values. Percentages for action choice for TBI and healthy controls are shown in Table 1. The *X* indicates the course of action predicted by the model. As it can be observed, the results show no significant difference, with TBI patients performing slightly better than controls. Participants were also asked to pick out the emotions they would feel being in the characters' shoes. Table 2 depicts percentages for emotion selection for the Actor Studio testing methodology for both experimental groups. The X indicates those emotions to be included in the agent's emotional state according to the MEA model. As Table 2 shows, TBI participants tend to select a larger number of emotions in every scenario.

**Table 1:** Actor studio: percentages for action choice.

**Scenario**	**Plan**	**MEA**	**TBI (%)**	**Controls (%)**
1. Wallace	*giving_key*	*X*	46.2	18.2
	*refusing_key*	–	53.8	81.8
2. At school!	*revenging*	*X*	84.6	81.8
	*letting_go*	–	15.4	18.2
3. A difficult choice	*staying*	X	84.6	81.8
	*leaving*	–	15.4	18.2

**Table 2:** Actor studio: percentages for emotion choice.

	**Wallace**			**At school!**			**A difficult choice**		
**Emotion**	**M**	**TBI (%)**	**C (%)**	**M**	**TBI**	**C (%)**	**M**	**TBI (%)**	**C**
Distress	–	38.5	9.1	–	23.1	–	X	23.1	18.2
Joy	–	23.1	–	X	23.1	36.4	X	76.9	54.5
Fear	–	–	–	–	46.2	54.5	–	23.1	9.1
Hope	–	15.4	18.2	–	–	–	–	30.8	9.1
Pride	–	23.1	9.1	X	23.1	27.3	–	38.5	36.4
Shame	X	23.1	27.3	X	23.1	18.2	X	23.1	18.2
Admiration	–	23.1	27.3	–	7.7	–	–	30.8	18.2
Reproach	–	7.7	27.3	–	53.8	36.4	–	7.7	9.1
Gratification	–	7.7	18.2	X	53.8	–	–	53.8	27.3
Gratitude	–	–	–	–	7.7	–	–	23.1	9.1
Remorse	–	38.5	27.3	–	15.4	27.3	X	69.2	45.5
Anger	–	23.1	9.1	–	46.2	9.1	–	7.7	9.1

Table 3 illustrates score means for the Actor Studio testing methodology both for TBI participants and controls. We ran Mann–Whitney statistic (*p* < 0.05) in order to compare means; no statistically significant difference was found between groups. However, there are some differences that deserve further consideration: all scores are slightly higher for TBI patients than controls (for Action Total Score, Emotion Total Score, Moral Emotion Score, Non Moral Emotion Score), but at the same time, their error scores (Error Total Score, Moral Emotion Error Score, Non Moral Emotion Error Score) also have higher values. In order to explore whether the observed tendency of TBI participants to select a large number of (often inconsistent) emotions would effect their scores, we measured Simple Matching Coefficients. Before computing SMCs for each scenario, we calculated the global SMC for all scenarios included in the Actor Studio methodology, as we believed emotion choices could be investigated as a single array of selected (and unselected) emotion labels, to be compared with MEA model predictions. When considering the complete emotion set, the mean SMC was 0.71 for controls and 0.67 for TBI participants. Focusing on moral emotions only, mean was 0.67 for TBI participants and slightly higher (0.70) for controls. We compared SMC through Mann-Whitney statistic. No statistically meaningful difference was found for both global (U = 40.000; sig = 0.118) and moral (U = 53.000; sig = 0.449) emotion set. SMCs were then computed for each scenario separately and hence compared using Mann–Whitney statistic (sig. < 0.05); no statistically significant difference was found between groups. Table 4 shows Mann–Whitney statistic for SMCs for the Actor Studio methodology. SMCs were measured on the complete emotion set. Table 5 illustrates Mann-Whitney comparison on the SMCs of the two groups, calculated on moral emotions only.

**Table 3:** Actor studio: mean scores.

**Group**	**ATS**	**ETS**	**MES**	**N-MES**	**ERTS**	**MERS**	**N-MERS**
TBI	2.15 (0.081)	3.38 (0.768)	2.15 (0.081)	1.23 (2.934)	11.31 (2.934)	8.00 (2.160)	3.31 (1.109)
Controls	2.00 (0.632)	2.45 (2.067)	1.36 (1.567)	1.09 (0.831)	10.2 (2.832)	7.64 (2.111)	2.64 (1.027)

**Table 4:** Actor studio: simple matching coefficients (all emotions).

**Scenario**	**TBI**	**Controls**	**MW U**	**sig. (2-tailed)**
Wallace	0.76	0.82	67.500	0.811
At school!	0.57	0.60	66.000	0.743
A difficult choice	0.64	0.65	65.000	0.700

**Table 5:** Actor studio: simple matching coefficients (moral emotions).

**Scenario**	**TBI**	**CONTROLS**	**MW U**	**Sig. (2-tailed)**
Wallace	0.75	0.77	62.000	0.826
At school!	0.59	0.59	71.000	0.976
A difficult choice	0.66	0.69	67.5000	0.811

#### 4.2.2. Results Audience

Due to methodology constraints (see section 3.3), data were analyzed separately for each scenario. Subjects were asked to select those emotions they believed better described the emotional states of characters in the scenario. Tables 6, 7 illustrate percentages for emotion selection for the Audience Studio test for both experimental groups. The X indicates those emotions to be included in the agent's emotional state according to the MEA model. Percentages for the main character in the scenario are shown in Table 6; percentages for the character interacting with the protagonist are demonstrated in Table 7. With respect to the results of the Actor Studio test (see previous section, Table 2), the TBI patients' tendency to select a larger number of emotions here is more evident.

**Table 6:** Audience studio: percentages for emotion choice (Main character).

	**Hamlet**			**The count of MonteCristo**			**Thérése Raquin**			**The vicomte of bragelonne**		
**Emotion**	**MEA**	**TBI**	**C**	**MEA**	**TBI**	**C**	**MEA**	**TBI**	**C**	**MEA**	**TBI**	**C**
admiration	–	–	10.0%	–	21.4%	37.5%	–	–	–	–	46.2%	30.0%
reproach	X	46.7%	50.0%	–	28.6%	25.0%	–	30.8%	11.1%	–	7.7%	–
joy	–	–	–	X	35.7%	50.0%	–	–	11.1%	–	53.8%	70.0%
gratification	–	–	10.0%	X	42.9%	87.5%	–	–	11.1%	X	69.2%	60.0%
gratitude	–	–	20.0%	–	7.1%	–	–	–	–	–	7.7%	–
pride	–	–	10.0%	X	71.4%	75.0%	–	7.7%	11.1%	–	53.8%	60.0%
fear	–	13.3%	10.0%	–	21.4%	–	–	46.2%	55.6%	–	23.1%	10.0%
anger	X	100%	30.0%	–	28.6%	25.0%	–	30.8%	–	–	–	–
remorse	–	6.7%	10.0%	–	14.3%	–	X	76.9%	88.9%	–	15.4%	–
hope	–	0.0%	10.0%	–	35.7%	–	–	15.4%	–	–	46.2%	30.0%
distress	X	80.0%	30.0%	–	14.3%	–	X	69.2	44.4	–	15.4 %	–
shame	–	33.3%	–	–	14.3%	–	X	76.9	77.8	–	15.4 %	10.0%

**Table 7:** Audience studio: percentages for emotion choice (other character).

	**Hamlet**			**The Count of MonteCristo**			**Thérése Raquin**			**The Vicomte of Bragelonne**		
	**TBI**	**C (%)**	**MEA (%)**	**TBI**	**C (%)**	**MEA Emotion (%)**	**TBI**	**C (%)**	**MEA (%)**	**TBI**	**C (%)**	
Admiration	–	–	–	–	–	–	–	–	–	–	46.2	60.0
Reproach	-	20.0	20.0	–	35.7	–	–	30.8	33.3	–	–	–
Joy	–	–	–	–	–	–	–	–	11.1	–	76.9	60.0
Gratification	–	–	–	–	–	–	–	–	11.1	–	30.8	10.0
Gratitude	–	–	–	–	–	–	–	7.7	–	X	84.6	60.0
Pride	–	6.7	–	–	7.1	–	–	7.7	–	–	15.4	–
Fear	–	26.7	50.0	–	78.6	50.0	–	38.5	33.3	–	15.4	–
Anger	–	33.3	–	–	28.6	50.0	–	46.2	33.3	–	15.4	10.0
Remorse	–	53.3	40.0	–	57.1	25.0	X	76.9	55.6	–	7.7	10.0
Hope	–	6.7	20.0	–	– –	–	–	15.4	–	–	38.5	30.0
Distress	–	73.3	10.0	X	42.9	37.5	X	23.1	66.7	–	23.1	–
Shame	X	86.7	50.0	–	57.1	62.5	X	84.6	66.7	–	23.1	–

In order to better assess score differences for emotion selection, Simple Matching Coefficients were computed for each scenario for both TBI participants and controls: similarly to the Actor Studio test, the SMCs are higher for controls than TBI patients for all scenarios. SMCs (calculated considering the complete emotion set) were then compared through Mann–Whitney statistic (sig.<0.05). Table 8 illustrates results for Mann-Whitney comparison. Results for moral emotions are shown in Table 9. In both cases, no significant differences were found.

**Table 8:** Audience studio: simple matching coefficients (global emotion set).

**Scenario**	**Mann–Whitney U**	**Sig. (two-tailed)**
Hamlet	53.000	0.210
The Count of MonteCrist	45.000	0.449
Thérèse Raquin	53.000	0.711
The Vicomte of Bragelonne	49.500	0.542

**Table 9:** Audience studio: simple matching coefficients (moral emotion set).

**Scenario**	**Mann–Whitney *U***	**Sig. (two-tailed)**
Hamlet	55.500	0.262
The Count of MonteCrist	35.500	0.156
Thérèse Raquin	53.500	0.733
The Vicomte of Bragelonne	55.500	0.262

## 5. Discussion

In this work, the experiments described in section 3 were carried out with a group of patients dealing with the consequences of TBI and with a control group of neurologically intact individuals, to explore group differences in the understanding of characters' moral behavior and emotions. In fact, according to research, TBI patients often exhibit impairments in social cognition, despite undergoing effective rehabilitation. More precisely, our study taps into the domain of moral emotion recognition in stories, as an important yet often neglected (McDonald, 2013) aspect of social cognition. Furthermore, our work contributes to research on narrative understanding in brain damaged populations, as this topic, to the best of our knowledge, has not been thoroughly explored yet.

### 5.1. Group Differences

Emerging differences, although not significant, show a clear trend and provide useful insight on the underpinnings of the impairment in TBI individuals and on the use of emotional virtual agents for rehabilitation. We first measured percentages for both action and emotion choice for each scenario for the Actor Studio methodology. After this, participants' choices were transformed into scores, to allow group comparisons. Concerning Action choice, TBI participants performed slightly better than controls, specially in the first scenario (see Table 1). Nonetheless, these results came as no surprise, as controls had to become confident with the procedure by themselves, whereas TBI patients participated in the study with the help of the experimenter. We believe randomized scenarios would be necessary for future work. Despite this, Mann–Whitney testing revealed no significant difference between groups (sig. = 0.494). Emotion scores (both moral and non-moral) are higher for the clinical sample: although unexpected (according to our hypothesis), this can be explained by observing error scores. As the testing methodology required participants to pick out emotions from a list and emotion scores were computed assigning 1 for each correct emotion, those being unselective (i.e., picking out a large number of emotions, although inconsistent) could have indeed scored higher, as their probability to guess the correct emotion was increased. In fact, for both moral and non-moral emotions, error scores are lower for controls, suggesting a link between TBI patients' better performance and their tendency to be unselective when judging emotions (as percentages for emotion choice demonstrate).

Moreover, we observed the results obtained by measuring the Simple Matching Coefficient (SMC) for each scenario and for the Actor Studio methodology as a whole (see Tables 4, 5). In fact, although it helps explaining group differences, error score fails in providing thorough information on the mismatch (or accordance) between the agent model and participants' expectations. SMC, on the other hand, allow measuring similarity between the agent model and participants' expectations more completely. We then compared SMCs through Mann–Whitney statistics. Mann-Whitney testing has shown no statistically significant difference for both complete and moral-only emotion set (U = 40, sig. = 0.118; U = 53, sig. = 0.449). Interestingly, we found a pattern in means for SMCs: although Mann-Whitney statistic did not show any significant difference, controls performed better than TBI participants for each scenario. It has to be noticed that the protocol required TBI participants to be not impaired in their ability to read and understand the narrative content of the stories, along with having acceptable attention span and visual abilities. As they did not differ in their understanding of the narrative content of the stories, we believe the SMC pattern we found (with TBI participants' SMC being lower than controls' for each scenario) might be ascribed to a specific impairment for the emotional content of the scenarios. As the slight differences we found seemed specific for emotion tasks, we think the action choice, where participants had to guess the course of action characters would choose according to their moral values, leverages reasoning abilities rather than emotional skills.

We further analyzed how participants evaluated characters' emotions. Regarding this, results for the Audience Studio testing methodology might help explain the score difference found for the Actor Studio methodology. As Tables 6, 7 show, TBI patients often selected the correct emotion, although percentages for the emotion predicted by the model were mostly higher for controls. Nevertheless, what the results for the Audience Studio help illustrate, is the fact that TBI participants tended to be unselective toward emotions, seeming somewhat puzzled from the task. For instance, in “The Count of Montecristo” all emotions were selected at least once by TBI participants, in some cases strongly inconsistently with MEA model predictions. Although the same situation, according to appraisal theories, can be appraised from different perspectives, resulting in different emotions to be generated for the same situation, some possibilities are ruled out by the model of emotional appraisal embedded in the agent. Finally, we compared group means of the Simple Matching coefficients for each scenario, using Mann-Whitney statistic. We found no statistically significant difference (see Tables 8, 9), both for complete and moral emotion sets. Further research may help outline dissimilarities between TBI patients and healthy controls; to do so, however, larger samples are needed.

### 5.2. Moral Emotion Understanding in Stories After TBI

Whereas not many works have addressed moral understanding in stories, literature (Greene and Haidt, 2002; Ciaramelli et al., 2007; Greene, 2015) illustrates how brain damaged individuals differ from neurologically intact participants for moral personal dilemmas. Personal moral dilemmas are dilemmas that elicit emotional responses while evaluating the dilemma, whereas non-personal and non moral dilemmas trigger no emotional response. When facing a moral personal dilemma, TBI participants differ from controls whilst they don't seem to differ when the dilemma elicits no emotion. According to Greene and Haidt (2002) personal moral dilemmas activate medial front gyrus, superior frontal gyrus while non-personal moral dilemmas engage dorsolateral and prefrontal areas; the same pattern can be observed when evaluating non-moral dilemmas. As Moretti et al. (2009) point out, TBI individuals whose brain damage involves areas such as the ventromedial prefrontal cortex can perform moral judgments (in terms of right/wrong), but show impairments in the emotional counterpart, in what is often referred to as “moral emotions selective impairment.” Furthermore, Hutcherson et al. (2015) illustrated the interplay between utilitarian and emotional appraisal during moral judgment based on moral values: utilitarian and emotional moral appraisals are computed separately and later integrated in a moral value response. Furthermore, TBI individuals seem to exhibit a particularly utilitarian pattern of judgments in moral dilemmas (Rowley et al., 2018). In our view, the emotional appraisal is the one of the reasons behind the impaired performance we observed in TBI patients, resulting in non standard moral judgments. Although not significant, the “selective impairment” we found for the emotional content of the scenarios might be attributed to the aforementioned deficits.

### 5.3. Implications for Agent-Based Rehabilitation

Taken altogether, our results further demonstrate the suitability of the MEA model (Battaglino et al., 2013) for developing emotional virtual agents and characters, and for their use in rehabilitation. The difference we found is, in fact, what could be predicted according to research on TBI participants, but also what we could notice by interacting with them. For example, *A*'s performance seems to be paradigmatic of the overall results we found. *A*'s incident had occurred 10 years before the testing took place, and she had spent 9 into rehabilitation. She was able to read and understand the scenarios thoroughly and she was very interested in and entertained by reasoning upon the emotional content of the stories. When asked to predicted the characters' action, she scored 2 out of 3, as most controls did, whereas she only scored 3 out of 9 when asked about the emotions. As she herself pointed out during the testing procedure, emotions are the hardest part of her outcome, only understandable when “black or white”; she perceives the gap between her current limitations in dealing with emotions and her condition before the trauma and would like to regain her full capability to understand emotions.

We also had the goal to collect preliminary data for the development of a rehabilitative tool in which patients face a moral situation, addressing their ability to perform moral reasoning and reasoning on their moral emotions through a virtual agent, following the paradigm discussed in Lisetti and Hudlicka (2014) and Hudlicka (2016). We investigated the correlation between the SMCs of the emotions selected by the TBI patients in the Actor Studio test and the rehabilitation duration or the time distance since the trauma. Remember that, as described in section 4, we leveraged SMCs to evaluate the similarity of the emotions selected by the patients for each character with respect to the predictions established by the MEA agent. We performed Spearman correlation on SMCs of the patients in the Actor Studio test and the duration of the rehabilitation (in years), finding no meaningful correlation (*r* = −0.001 *p* = 0.996; two-tailed). As the rehabilitation the patients were undergoing did not include moral emotions or moral scenarios, this result came as no surprise. Similarly, we found Simple Matching Coefficients to be unrelated to time distance as well (*r* = 0.125 *p* = 0.699; two tailed). In this, the results obtained were promising for the design of agent-based rehabilitation tools, since they show that, in the absence of a specific rehabilitation targeted on emotions, and moral emotions in particular, no advancements are obtained.

On the one side, our experiments confirm the feasibility of this type of rehabilitation, further demonstrated by the comments of our TBI participants (already reported in Ceccaldi et al., 2016): *A*—“this test allowed me to think about the values that shape my moral judgment, helping me understand how every action comes after deep and elaborate reasoning”; *R*—believes this test “is important, as it makes my head start again.” On the other side, the indirect validation of the MEA model provided by the study opens the way to the use of virtual agents in rehabilitative settings, as it contributes to the specification of an accountable agent model for developing practical applications.

## 6. Conclusion

In this paper, we described and discussed the results of the experiments on emotion understanding in stories that we conducted on a group TBI patients by leveraging a moral emotional agent. In brief, taking the agent model as a reference, we compared emotion recognition in TBI individuals and in a control group of neurologically intact participants. The results show that, notwithstanding no statistically meaningful difference between the two groups, the performance of the TBI patients differed from the control group concerning moral emotions. The main finding of our work was that the difference between the two groups was far from significant for those dimensions pertaining to the characters' course of action, whereas scores were higher for controls than for TBI participants for each scenario and for both test types. TBI participants performed differently from controls when asked to evaluate the emotional content of narrative scenarios. A complete and thorough definition of all the features that differentiate impaired moral and non-moral emotion understanding in people who suffered from TBI from that of healthy controls is far from achieved. Nonetheless, our results demonstrate the need to focus on emotion processing (rather than on reasoning) in order to gain a deeper understanding. This is in line with the mental models theory perspective on moral reasoning (Bucciarelli et al., 2008). More precisely, according to this perspective, moral reasoning is regular reasoning that happens to concern moral issues. The TBI patients included in our study were in a late stage of their rehabilitation; as a consequence, their reasoning could be compared to those of neurologically intact individuals. Despite this, their performance varied more significantly from that of controls for those tasks requiring (moral or non-moral) emotional processing. In our opinion, having patients whose outcome is similar to our participants' interacting with virtual characters whose emotional state holds a moral component could be effective in helping these patients improve their emotional moral functioning and their understanding of moral (but also non-moral) emotions in characters. Conversely, we believe also that modeling “impaired” moral characters would provide effective and useful agent-based rehabilitative applications and more engaging virtual environments.

## Data Availability Statement

The datasets generated for this study are available on request to the corresponding author.

## Ethics Statement

Ethical review and approval was not required for the study on human participants in accordance with the local legislation and institutional requirements. Written informed consent for participation was not required for this study in accordance with the national legislation and the institutional requirements.

## Author Contributions

All authors listed have made a substantial, direct and intellectual contribution to the work, and approved it for publication.

## Conflict of Interest

The authors declare that the research was conducted in the absence of any commercial or financial relationships that could be construed as a potential conflict of interest.

## Footnotes

^1^https://ec.europa.eu/info/sites/info/files/commission-white-paper-artificial-intelligence-feb2020_en.pdf

^2^Puzzle rehabilitation center is an Italian leading facility for neuropsychological recovery after traumatic brain-injury (www.centropuzzle.org/).
