# Supplementary material for: An Emotional Agent for Moral Impairment Rehabilitation in TBI Patients
Source: Front Psychol. 2020 Jun 30;11:1102. doi: 10.3389/fpsyg.2020.01102 (PMC7338226; doi:10.3389/fpsyg.2020.01102)
Supplement: Supplementary file 1 [file Data_Sheet_1.pdf]

Supplementary Material

1 APPENDIX

1.1 Actor Studio

Table S1. Scenarios for Actor Studio test

Scenario	Character	Plan	Values at stake	Values balanced	Emotions
1. Wallace and uncle George's roses	Wallace	giving$_{k}ey$	Honesty	–	Shame
	Wallace	refusing$_{k}ey$	Loyalty	–	Shame
2. At school!	Tom	revenging	Pity	Justice	Shame, Joy, Pride (Gratification)
	Tom	letting$_{g}o$	Justice	–	Anger
3. A difficult choice	Mark	staying	Family	–	Joy, Shame, Distress (Remorse)
	Mark	leaving	Happiness	–	Joy, Shame, Distress (Remorse)

Scenario 1. Wallace and uncle George's roses. This scenario is based on the 'nunnery scene' in Shakespeare's Hamlet. Ophelia, has to decide whether to lie to the protagonist, Hamlet, thus putting at stake her 'Honesty', or to reveal the truth, putting at stake the 'Loyalty' towards her father. *Wallace and Charlie are cousins. They live in the countryside where uncle George has a nursery of precious roses he brings to gardening contests. Uncle George is very jealous of his roses. Charlie wants to make a gift to his girlfriend and asks Wallace to give him the key of the nursery to get one. Wallace has to choose whether to be loyal to his cousin Charlie or to uncle George, but loyalty has a higher priority than honesty.* The agent finds two plans:

- the plan $giving\_key$ consists of giving the key to Charlie, thus deceiving uncle George, and puts at stake the value *Honesty*, so Wallace will feel *Shame*;
- the plan $refusing\_key$ contains the action of refusing to give the key to Charlie and puts at stake the value *Loyalty* to Charlie (which has a higher priority than *Honesty*), so Wallace will again feel Shame (here, for putting at stake his loyalty).

Assuming the same probability of success, the anticipatory emotional appraisal leads Wallace to choose $giving\_key$ since the Shame intensity is lower.

Scenario 2. At school!. This scenario is based on the situation termed 'Crime pursued by vengeance' from the catalog of 36 dramatic situations compiled by **?**.

This situation depicts the vengeance as a divine Joy felt by those who pursue it after being victim of a crime with no guilt. *Tom is bullied by his classmate Pier, who has taken from Tom the role of director of the school newspaper, putting around lies about him. Tom notices that Pier has forgotten his backpack with all his stuff in the locker room. Tom digs in Pier's backpack and finds evidence that Pier copied the class test. Tom is now uncertain about what to do, whether to take revenge against Pier or to pass through this situation. Tom has to choose whether he wants to take vengeance or not*. The MEA agent finds two plans:

- the plan $revenging$ contains the action of humiliating Pier and puts at stake the value *Pity*, so Tom will feel Shame, but it also brings back to balance the value *Justice* (the value with the highest priority) put at stake by Pier; moreover, this plan satisfies the goal of being the director again, so Tom will feel

Joy (for satisfying his goal) and *Pride* (for restoring his value *Justice*), which yield the compound *Gratification* emotion;

- the plan $letting\_go$ contains the action of letting it go and has no effects on Tom's goals and values: if Tom performs this plan, the situation doesn't change and the value *Pity* is not put at stake: since the value *Justice* was put at stake by Pier before, Tom still feels *Anger* towards Pier.

Assuming the same probability of success, Tom chooses to execute the plan $revenging$ because of the higher priority of justice to him: he will feel *Shame* for putting at stake the value *Pity*, but will also feel *Gratification* (as *Joy* and *Pride*).

Scenario 3. A difficult choice. This scenario is based on the 'Remorse' in Polti's catalog **?**, in which the culprit feels Remorse for an evil deed. *New York, 2003. Mark and Lucy are married and have a beautiful baby. Lucy has agreed to spend a couple of years in Italy for the job of her dreams: working as a curator of a famous art gallery. Mark, however, has always wanted to be judge in New York. Just when Lucy has officially accepted her job in Italy, Mark gets the seat as a judge in New York. Now, Mark has to choose whether to have the job or to stay with his family.* The MEA agent finds two plans:

- the plan $staying$, which consists of staying in New York, satisfies Mark's goal of being a judge and threats the goal of being with the family, thus putting at stake the value *Family*. He will feel Joy for satisfying his goal of being a judge, but also Shame (for putting at stake a value) and *Distress* (for threatening the goal of being with the family), which combine into the emotion of *Remorse*.
- the plan $leaving$ consists of going to Italy, which satisfies the goal of being with the family but puts at stake the goal of being a judge and the value *Happiness* (which has a higher priority than *Family* for Mark), so, again, Mark will feel *Shame* and *Distress*, but also *Joy* for satisfying his goal of being with the family.

The agent chooses the plan $staying$ since it puts at stake the value with the lower priority, *Family*, due to the equal importance of success of both goals: in any case, Mark would feel *Shame*, but the *Remorse* will be lower for $staying$.

1.2 Audience Studio

Table S2. Scenarios for Audience Studio test

Scenario	Character	Plan	Values at stake	Values balanced	Emotions
1. Hamlet	Hamlet	–	Honesty	–	Reproach, Distress, Anger
	Ophelia	*lying*	Honesty	Loyalty	Shame
2. The Count of Monte Cristo	Edmond	*taking_revenge*	–	Justice	Joy, Pride, Gratification
	Fernand	–	–	–	Distress
3. Thérèse Raquin	Thérèse	*killing*	Honesty	–	Distress, Reproach, Remorse
	Camille	*killing*	Honesty	–	Distress, Reproach, Remorse
4. The Vicomte of Bragelonne	Aramis	*freeing*	Freedom	–	Pride, Joy, Gratification
	Philippe	–	Freedom	–	Admiration, Joy, Gratitude

Scenario 1. Hamlet, W. Shakespeare. The scenario is the so-called "nunnery scene". *Ophelia is sent to Hamlet by Polonius and Claudius to confirm the assumption that his madness is caused by his rejected love. Hamlet asks Ophelia where her father is to test her honesty, knowing that he is hiding in the room;*

she answers with a lie. The goal of Hamlet is to save Ophelia from the corruption of the court: asking her about her father is a way to test her honesty (value *Honesty*), since he knows that Ophelia's father is spying them. Ophelia has the moral value *Honesty* as well, but also the *Loyalty* value towards her father. Ophelia answers with a lie, saying that her father is at home. The *lying* plan is appraised by Hamlet as blameworthy because it puts his value at stake, so he feels *Reproach*. At the same time, Ophelia's *lying* plan makes Hamlet's goal (saving her) not achievable, so Hamlet feels *Distress*, which yields *Anger* combined with Reproach. Ophelia feels *Shame* for being dishonest with Hamlet, because she sets the value *Honesty* aside in favor of the *Loyalty* to her father.

Scenario 2. The Count of Monte Cristo, Alexandre Dumas. This scenario is about Edmond Dantes' revenge on Fernand. *Edmond, disguised as the Count of Monte Cristo, finds evidence of Fernands' crime and devises a plan to get revenge. Fernand challenges Edmond to a duel and Edmond reveals his true identity to Fernand.* Edmond has the goal to get revenge and his value *Justice* is put at stake by his imprisonment. The plan of *taking_revenge* brings Edmond's value back to balance, achieving at the same time his goal to take revenge, so he feels *Gratification* (*Joy* and *Pride*).

Fernand feels *Distress*, because his goal to keep his social status has failed.

Scenario 3. Thérèse Raquin, E. Zola's . Thérèse, is unhappily married to her cousin, Camille, and has a sordidly passionate affair with one of Camille's friends, Laurent. The two lovers decide to kill Camille by drowning him during a boat trip, but the remorse wears out their love day by day. *Thérèse and Laurent live in the remorse of murdering Camille.* Thérèse and Laurent have executed a *killing* plan that put at stake one of their values, *Honesty*; so, they feel *Reproach*. *Killing* has also made their goal of being happy together not achievable anymore, so they feel *Distress*, which generates the compound emotion *Remorse* (*Reproach* and *Distress*). **Scenario 4. The Vicomte of Bragelonne, A. Dumas**. Philippe, the twin brother of Louis XIV is imprisoned from birth by his parents. *The scenario depicts the moment in which Aramis frees Philippe from the prison.* By freeing Philippe (*freeing* plan), Aramis brings back to balance his value of *Freedom*, achieving at the same time the goal of setting Philippe free; Philippe has the same goal and value. So, Aramis feels *Gratification* (*Pride* and *Joy*) for his own action.

Philippe feels *Gratitude* (*Admiration* and *Joy*) towards his savior.
